# Should we restrict erythrocyte transfusion in early goal directed protocols?

**DOI:** 10.1186/s12871-015-0054-4

**Published:** 2015-05-09

**Authors:** Patrick Meybohm, Aryeh Shander, Kai Zacharowski

**Affiliations:** 1 Department of Anaesthesiology, Intensive Care Medicine and Pain Therapy, University Hospital Frankfurt, Theodor-Stern-Kai 7, 60590 Frankfurt am Main, Germany; 2 Department of Anesthesiology and Critical Care and Hyperbaric Medicine, Englewood Hospital and Medical Center, Anesthesiology and Critical Care and Hyperbaric Medicine, Englewood, NJ USA

**Keywords:** Allogeneic blood transfusion, Protocol directed therapy

## Abstract

**Background:**

Early goal-directed therapy has been endorsed in the guidelines of the Surviving Sepsis Campaign as a key strategy among patients presenting with severe sepsis or septic shock. But more importantly, early goal-directed therapy also became standard care for non-septic critically ill patients and was adopted for high-risk surgical patients.

**Discussion:**

Importantly, transfusion of red blood cells is a central part of many protocols of early goal-directed therapy to indicate the need for use of inotropes and red blood cells, as both central venous saturation and hematocrit are used as transfusion triggers. However, burgeoning data has strongly linked transfusion with worse clinical outcomes. If correct, could these early goal-directed therapy ‚bundles’ have better outcome if a restrictive transfusion practice is adopted?

**Summary:**

Early goal-directed therapy has evolved as standard care for most of critically ill patients, and many protocols contain transfusion of red blood cells targeting high hemoglobin level as a key element. As red blood cell transfusions are associated with increased morbidity and mortality, transfusion thresholds need to be more individualized.

## Background

In a single-center study published in 2001 involving patients presenting with severe sepsis and septic shock, mortality was markedly lower among those who were treated according to a 6-hour protocol of early goal-directed therapy (EGDT) than among those receiving usual care [1]. The EGDT protocol included indications for central venous pressure and mean arterial pressure (<65 mm Hg) to guide both fluid and vasopressor administration. Additionally, both central venous saturation (<70%) and hematocrit (<30%) were included to indicate the need for use of inotropes and red blood cell (RBC) transfusion, respectively.

Importantly, burgeoning data has strongly linked transfusion with worse clinical outcomes. If correct, could these EGDT ‚bundles’ have better outcome if a restrictive transfusion practice is adopted?

## Discussion

Following these auspicious results [1] EGDT has been endorsed in the guidelines of the Surviving Sepsis Campaign as a key strategy among patients presenting with severe sepsis or septic shock. But more importantly, EGDT also became standard care for non-septic critically ill patients and more recently, EGDT was adopted for high-risk surgical patients [2]. Thus, protocols were implemented at hospitals around the world incorporating all elements of the care bundle, but the impact of the different elements of an EGDT protocol has yet to be individually investigated. Based on current knowledge, it may be conceivable that individual elements may even be harmful (e.g. RBC transfusion and central venous pressure), and thereby reducing the potential of more beneficial effects.

In this respect, we would like to caution the reader:

In previous EGDT protocols transfusion of RBC targeting hemoglobin >8 g/dL or hematocrit level > 30% is a key element to increase central venous oxygen saturation (Table 1). Importantly, evidence for augmentation of oxygen delivery and thereby increase of central venous oxygen saturation above 70% by RBC transfusion is poor. In contrast, a rational use of RBC concentrates is mandatory, as RBC transfusions are associated with increased morbidity and mortality [3].

**Table 1:** **Overview of early-goal directed protocols and high transfusion triggers (selection)**

Study	Field of interest	RBC transfusion trigger
Rivers et al. [1]	Severe sepsis and septic shock	Hct < 30%, if ScvO_2_ < 70%
Lobo et al. [10]	Major non-cardiac surgery	Hct < 30%, if PAOP < 16 mm Hg
Donati et al. [11]	Major abdominal surgery	Hb < 10 g/dl, if CVP < 10 mm Hg
Smetkin et al. [12]	Cardiac surgery	Hb < 8 g/dl, if ScvO_2_ < 60%
ProMISe Investigators [13]	Severe sepsis and septic shock	Hb < 10 g/dl, if ScvO_2_ < 70%
ProCESS Investigators [4]	Septic shock	Hct < 30%, if ScvO_2_ < 70%
ARISE Investigators [5]	Septic shock	Hct < 30%, if ScvO_2_ < 70%

Hct indicates hematocrit; Hb, hemoglobin; ScvO_2_, central venous oxygen saturation; CVP, central venous pressure; PAOP, pulmonary artery occlusion pressure.

In the recent ProCESS trial [4] 1,341 patients with septic shock were randomly assigned to one of three groups for 6 hours of resuscitation: protocol-based EGDT including RBC transfusion if hematocrit < 30% and central venous saturation < 70%; protocol-based standard therapy or usual care. The primary end point was 60-day in-hospital mortality. Not surprisingly, patients in the EGDT group compared to usual care received significantly more vasopressors (54.9% vs. 44.1%, P = 0.003), more dobutamine (8.0% vs. 0.9%; P < 0.001), and more RBC transfusion (14.4% vs. 7.5%; P = 0.001) without any clinical benefit but used more resources.

In the more recent ARISE trial [5] 1,600 patients with early septic shock were randomly assigned to receive either EGDT or usual care. The primary outcome was all-cause mortality within 90 days after randomization. Again, patients in the EGDT group were more likely to receive vasopressor infusions (66.6% vs. 57.8%; p < 0.001), dobutamine (15.4% vs. 2.6%; p < 0.001), and RBC transfusion (13.6% vs. 7.0%; P < 0.001) again with very similar outcomes as ProCESS.

Both studies confirmed the most important elements in management of sepsis: early administration of antibiotics and early adequate volume resuscitation using clinical parameters ascribed by the protocol. Of note two of the areas questioned here, the indication for dobutamine and transfusion “triggers” of hematocrit < 30% must be reassessed in light of existing evidence.

RBC transfusions are frequently given to patients with septic shock. Few of these transfusions are given to patients who are bleeding and most to non-bleeding patients. However, the use of a high hemoglobin threshold for transfusion as part of an EGDT protocol should be questioned. In this respect, Holst et al. [6] compared two different transfusion strategies and randomized 1,005 patients with septic shock to receive one unit RBC when the hemoglobin level was ≤7 g/dl (lower threshold) or when the level was ≤9 g/dl (higher threshold) during the ICU stay. Primary endpoint was mortality at 90 days that was similar between both groups. However, the lower-threshold group received a median of 1 unit of blood (interquartile range, 0 to 3) and the higher-threshold group received a median of 4 units (interquartile range, 2 to 7). These authors concluded that RBC transfusion at a hemoglobin threshold of 7 g/dl is safe in septic patients, and a higher threshold was not beneficial and resulted in a 10–20 times higher transfusion adverse events.

In high-risk cardiac surgical patients, Murphy et al. [7] recently randomly assigned 2,007 patients post cardiac surgery (they were either revasculerized or replaced defective valves) to a restrictive transfusion threshold (hemoglobin level <7.5 g/dl) or a liberal transfusion threshold (hemoglobin level <9 g/dl) group. The primary outcome was a serious infection (sepsis or wound infection) or an ischemic event (permanent stroke, myocardial infarction, infarction of the gut, or acute kidney injury) within 3 months after surgery. Transfusion rates were 53.4% (higher than many liberal transfusion hospitals) and 92.2% in the two groups, respectively. The restrictive transfusion threshold was not inferior to the liberal threshold with respect to morbidity or 30 day mortality. Mortality at 90 days was statistically higher in the restrictive group with 16 more deaths than in the liberal-threshold group. These finding are perplexing since the causes of death were not related to anemia and no plausible mechanism was offered by the authors. In this respect, this study provides non-inferiority data on restrictive transfusion and should not result in change of practice until these findings are either corroborated or refuted. It is also unclear whether the liberal transfused group were benefiting from the ‘volume therapy’ which the restrictive group has not received.

In-line with this discussion, two US health care organizations (American Medical Association Physician Consortium for Performance Improvement® and The Joint Commission) have previously recommended strategies to minimize overuse in healthcare, naming blood products as one of the top five targets. In addition, the Choosing Wisely® campaign launched by the American Board of Internal Medicine Foundation both in US and now Canada, repeat the same message [8]. The World Health Organization has adopted resolution 63.12, also adopted by the United States Department of Health and Human Services, recommending all member states to implement a patient blood management (PBM) program employing multiple strategies to minimize unnecessary exposure to blood products as a new standard of care. In detail, PBM is a proactive evidence based approach to identify, diagnose and treat anemia before a transfusion threshold is met. Optimization of hemostasis and minimization of blood loss are additional core principles of PBM to reduce costs and to improve patient outcome [9].

## Summary

EGDT has evolved as standard care for most of critically ill patients, and many EGDT protocols contain transfusion of RBC targeting hemoglobin >8 g/dL or hematocrit level > 30% as a key element to increase central venous oxygen saturation. In contrast, RBC transfusions are associated with increased morbidity and mortality, and therefore, a PBM program to minimize unnecessary exposure to blood products could be adopted within EGDT protocols. Additionally, transfusion thresholds for RBC transfusion need to be more individualized.

## Abbreviations

EGDT: Early goal directed therapy
PBM: Patient blood management
RBC: Red blood cell
